# Tacrolimus Trough Concentration‐Escalating Bipartite Therapy (Glucocorticoids Combined With Tacrolimus) for Refractory *MDA5* Antibody‐Positive Clinically Amyopathic Dermatomyositis With Rapidly Progressive Interstitial Pneumonia

**DOI:** 10.1002/iid3.70295

**Published:** 2025-11-05

**Authors:** Anji Xiong, Jiang Shao, Yanzao Zhao, Xuemei Huang, Jie Luo, Xi He, Pu Chen, Yiman Ye, Chuanmei Xie, Xin Wei, Xiongyan Luo

**Affiliations:** ^1^ Department of Rheumatology and Immunology Neijiang Sichuan China; ^2^ Department of Rheumatology and Immunology, Beijing Anzhen Nanchong Hospital Capital Medical University & Nanchong Central Hospital, The Affiliated Nanchong Central Hospital of North Sichuan Medical College Nanchong Sichuan China; ^3^ Department of Rheumatology and Immunology The Affiliated Hospital of Southwest Medical University Luzhou Sichuan China; ^4^ Department of Rheumatology and Immunology The Affiliated Hospital of North Sichuan Medical College Nanchong Sichuan China; ^5^ Department of Ophthalmology West China Hospital, Sichuan University Chengdu Sichuan China; ^6^ Department of Rheumatology and Immunology West China Hospital, Sichuan University Chengdu Sichuan China

**Keywords:** dermatomyositis, interstitial pneumonia, MDA‐5, tacrolimus, treatment

## Abstract

**Objective:**

This study aimed to determine the outcomes of patients that have refractory anti‐melanoma differentiation‐associated gene 5 (*MDA5*) antibody‐positive clinically amyopathic dermatomyositis with rapidly progressive interstitial pneumonia treated with tacrolimus trough concentration‐escalating bipartite therapy (glucocorticoids combined with tacrolimus).

**Methods:**

We retrospectively enrolled 19 patients that had refractory anti‐*MDA5* antibody‐positive clinically amyopathic dermatomyositis with rapidly progressive interstitial pneumonitis who were unable to achieve disease control on intensive combination immunosuppressive therapy with glucocorticoids and immunosuppressive agents. They were treated with tacrolimus trough concentration‐escalating bimodal therapy. Patients receiving tacrolimus trough concentration‐escalating dual therapy were selected based on poor prognostic factors and refractoriness to the initial immunosuppressive therapy.

**Results:**

The 19 patients included in the study were treated with tacrolimus trough concentration‐escalating bipartite therapy (glucocorticoids combined with tacrolimus) immediately after diagnosis of refractory anti‐melanoma differentiation‐associated gene 5 (*MDA5*) antibody‐positive clinically amyopathic dermatomyositis with rapidly progressive interstitial pneumonia. Following 17 months of treatment and subsequent follow‐up, 16 patients displayed decreased ferritin and lactate dehydrogenase levels and improved oxygenation index and high‐resolution lung computed tomography findings. Two patients died, and one patient (case 9) developed renal impairment on Day 14 of treatment. Renal damage occurred when the tacrolimus trough concentration was 15.68 ng/mL. After lowering the tacrolimus trough concentration, the patient′s renal function gradually normalized. However, because of the addition of cyclophosphamide due to disease activity, the patient did not experience the full efficacy of the two‐combination therapy. Tacrolimus‐associated renal impairment and viral and fungal infections occurred in some patients treated with bimodal therapy, but it did not have serious consequences.

**Conclusion:**

For patients with refractory anti‐*MDA5* antibody‐positive clinically amyopathic dermatomyositis associated with rapidly progressive interstitial pneumonia, the combination of glucocorticoids and tacrolimus is a potentially effective treatment regimen. However, there is considerable individual variation in the optimal trough concentration of tacrolimus.

## 1 Introduction

Anti‐melanoma differentiation‐associated gene 5 (*MDA5*)‐antibody‐positive clinically amyopathic dermatomyositis (CADM) is a special subclass of dermatomyositis (DM) characterized by typical skin lesions of DM without or with only mild muscle involvement and a very high mortality rate due to rapidly progressive interstitial lung disease (RPILD) [1, 2]. Intensive combination immunosuppressive therapy with glucocorticoids and immunosuppressive agents has been used to treat patients with anti‐*MDA5* antibody‐positive CADM with rapidly progressive interstitial pneumonitis [3]. However, the disease may remain uncontrolled despite intensive therapy [4]. Therefore, a new and effective treatment is needed for refractory anti‐*MDA5* antibody‐positive CADM in patients with rapidly progressive interstitial pneumonia whose disease is not controlled by intensive treatment.

Treatment with tacrolimus improves survival and further improves prognosis in patients with refractory DM with interstitial pneumonitis; thus, tacrolimus is recommended for such refractory patients [5, 6]. Furthermore, previous clinical trials have shown that tacrolimus has a dose‐dependent effect in patients with ulcerative colitis, with higher remission rates observed after increasing the dose of tacrolimus [7]. For patients with refractory anti‐*MDA5* antibody‐positive CADM and rapidly progressive interstitial pneumonia who are positive for anti‐*MDA5* antibodies, tacrolimus trough concentration‐escalating dual therapy (glucocorticoids combined with tacrolimus) is a therapeutic strategy worth considering.

Herein, we analyzed 19 patients with refractory anti‐*MDA5* antibody‐positive CADM with rapidly progressive interstitial pneumonitis treated with tacrolimus trough concentration‐escalating dual therapy (glucocorticoids combined with tacrolimus) because of a lack of disease control with intensive combination immunosuppressive therapy (glucocorticoids combined with immunosuppressive agents).

## 2 Methods

### 2.1 Patients

This retrospective study included 19 patients that had refractory anti‐*MDA5* antibody‐positive CADM with rapidly progressive interstitial pneumonitis who presented to Nanchong Hospital of Beijing Anzhen Hospital affiliated with Capital Medical University between September 2019 and February 2024. Rheumatologists use the diagnostic criteria proposed by Sontheimer and Gerami et al. [8, 9] to diagnose anti‐*MDA5* antibody‐positive CADM. It is a subclass of idiopathic inflammatory myopathies characterized by the hallmark cutaneous manifestations of DM, the absence of any clinical or laboratory evidence of muscle disease for ≥ 6 months, and a positive *MDA5* antibody test. Refractoriness was defined as the failure to respond to treatment with glucocorticoids and at least one immunosuppressive agent within 1 month [10, 11]. Patients with reticular clouding, ground‐glass opacities, or a honeycomb appearance were considered to have interstitial lung disease. The diagnosis of RPILD was made when the following conditions were met: worsening exertional dyspnea over several days to 3 months, a decline in spirometry, and an increase in fibrosis on high‐resolution computed tomography (HRCT) [12, 13]. A serum ferritin level > 1000 ng/mL before tacrolimus trough concentration‐escalating dual therapy, the presence of ground‐glass shadows in all six lung fields and worsening pulmonary infiltrates during the initial immunosuppressive therapy were associated with poor prognosis. The study was conducted following the principles of the Declaration of Helsinki and its amendments. Each patient provided written informed consent before enrollment. The study protocol was approved by the Medical Ethics Committee of Nanchong Hospital of Beijing Anzhen Hospital Capital Medical University (Ethical Review No. 2024 [038]).

### 2.2 Assessment Method for HRCT Improvement

Patients′ imaging evaluations were conducted via high‐resolution chest CT (HRCT), primarily involving qualitative assessments by radiologists based on imaging changes (e.g., reduction in ground‐glass opacities, absorption of consolidation areas). This study did not employ standardized scoring systems (e.g., Warrick score) for quantitative evaluation of imaging improvement. Future research may consider adopting standardized radiological scoring systems to further enhance the accuracy and reproducibility of study results.

### 2.3 Renal Function Assessment

Renal function in this study was evaluated using KDIGO criteria, defining renal impairment as a creatinine level reaching 1.5 times or more the baseline value, or a significant increase in BUN. To comprehensively assess renal function changes during treatment, baseline and peak creatinine and BUN values during therapy are listed in Table 1.

**Table 1:** Patient′s characteristics at baseline and clinical course.

Case	Case 1	Case 2	Case 3	Case 4	Case 5	Case 6	Case 7	Case 8	Case 9	Case 10	Case 11	Case 12	Case 13	Case 14	Case 15	Case 16	Case 17	Case 18	Case 19
Age/sex	59/F	50/M	48/M	58/M	48/F	51/F	48/M	57/F	52/M	47/F	50/F	36/M	52/M	52/F	52/F	55/F	56/M	66/F	63/F
Other comorbidities	—	—	—	—	—	—	—	—	—	—	—	—	—	—	—	—	Mediastinal emphysema	Mediastinal emphysema	—
Initial treatment	GC + TCZ+IVIg	GC+CsA	GC (Pulse therapy with 500 mg qd for three days)+ IVCY + TOF	GC + IVCY	GC (Pulse therapy with 1 mg/kg for three days) +IVCY+Baricitinib	GC + IVCY + TAC (Trough concentration of 1.96 ng/mL)	GC + IVCY + TAC (Trough concentration of 5.89 ng/mL)	GC + IVCY	GC + PE + TAC (Trough concentration of 7.11 ng/mL)	GC + TAC (Trough concentration of 0.69 ng/mL)	GC + TOF	GC + TAC (Trough concentration of 3.5 ng/mL) +Baricitinib	GC + MMF	GC + TOF + TAC (Trough concentration of 4.16 ng/mL)	GC + IVCY + TAC (Trough concentration of 3.66 ng/mL)	GC + TAC (Trough concentration of 5.94 ng/mL)	GC + IVCY+CsA	GC+Baricitinib	GC+IVIgTAC (Trough concentration of 6.25 ng/mL)
There was an increase in infiltration during the initial treatment regimen	+	+	+	+	+	+	+	+	+	+	+	+	+	+	+	+	+	+	+
Number of lung fields with GGO/consolation in patients before treatment with bipartite therapy	6	5	6	6	6	6	3	6	4	3	6	6	5	2	6	6	6	6	NA
Ferritin levels (ng/mL) in patients before bipartite therapy treatment	371	> 2000	> 2000	> 2000	> 2000	865.9	52.2	472	442	735	755	456	1789	> 2000	798	1050	> 2000	> 2000	> 2000
*Laboratory data (before bipartite therapy treatment/after bipartite therapy treatment)*																			
LDH (120.0–250.0 U/L)	656.8/332	572.5/276.2	711.2/209.2	582/210	421.9/221.2	540.7/227.2	421.9/221.2	382.8/242.3	268.8/219.1	287.9/220.8	310.3/256	215/238.5	365.8/253.4	261.8/163	340.3/229.1	249.1/189	607.7/425.8	424.9/524.5	728.5/572.3
Ferritin levels (M:30‐400 ng/ml F:13‐150 ng/ml)	371/273	> 2000/527	> 2000/380	> 2000/103	> 2000/56.5	869.5/36.1	> 2000/56.5	472/80.1	442/—	735/457	755/321	456/368	1978/923	> 2000/698.87	798/336	1050/663	> 2000/934	> 2000/ > 2000	> 2000/ > 2000
P/F ratio (mm Hg)	220/N	200/N	220/N	200/N	243/N	257/N	243/N	360/N	360/—	360/N	310/N	333/N	310/N	360/N	360/N	333/N	200/360	200/—	147.5/—
Tacrolimus optimal trough concentration (ng/mL)	15.86	17.57	19.84	15.11	17.45	9.39	17.45	8.05	NA (The patient developed renal damage at a tacrolimus trough concentration of 16.81 ng/mL and then normalized renal function when the tacrolimus trough concentration was lowered to 5.5 ng/mL)	8.01	10.01	14.58	6.97	15.68	23.7	16.72	36.41	NA	NA
Clinical outcomes of bipartite therapy treatment	Alive	Alive	Alive	Alive	Alive	Alive	Alive	Alive	Alive	Alive	Alive	Alive	Alive	Alive	Alive	Alive	Alive	Dead (Died of respiratory failure due to RP‐ILD.)	Dead (Died of respiratory failure due to RP‐ILD.)
Scr Before bipartite therapy treatment/after bipartite therapy treatment (μmol/L)	46/68	61/72	48/53	67/99	57/59	40/58	52/76	41/55	73/126	38/47	38/43	50/56	75/82	35/48	49/55	52/61	42/82	67/71	40/40
BU Before bipartite therapy treatment/after bipartite therapy treatment (mmol/L)	7.43/8.21	5.12/6.16	7.91/8.79	7.11/7.34	6.91/7.22	8.72/8.94	5.89/6.46	7.92/8.31	8.23/20.93	3.72/4.47	5.11/5.23	5.42/5.66	4.87/5.42	8.12/8.43	5.51/5.59	7.14/7.34	8.78/21.03	8.65/9.47	8.12/8.77
Adverse event	—	—	—	—	—	—	—	—	Renal damage	—	—	—	—	—	—	—	Renal damage, CMV reactivation, *Candida albicans* infection	CMV reactivation	—
Observation time for bipartite therapy	8 months	14 months	17 months	10 months	15 months	12 months	6 months	5 months	14 days (CY added due to disease activity after development of renal damage)	11 months	6 months	8 months	6 months	11 months	4 months	5 months	6 months	11 days	4 days

Abbreviations: BU, blood urea; CADM, clinically amyopathic dermatomyositis; CMV, cytomegalovirus; CsA, cyclosporine A; CY, cyclophosphamide; F, female; GC, glucocorticoids; GGO, ground‐glass opacity; IVCY, intravenous pulse cyclophosphamide; IVIg, intravenous immunoglobulin; IV CTX, intravenous Cyclophosphamide; LDH, lactate dehydrogenase; M, male; *MDA5*, melanoma differentiation‐associated protein 5; MMF, mycophenolate mofetil; N, normality; NA, not applicable; PE, plasma exchange; P/F ratio, PaO_2_/FiO_2_ ratio; RP‐ILD, rapidly progressive interstitial lung disease; Scr, serum creatinine; TAC, tacrolimus; TCZ, tocilizumab; TOF, tofacitinib.

### 2.4 Treatment Program

Patients were selected based on poor prognostic factors and refractoriness to the initial immunosuppressive therapy. Nineteen patients with refractory anti‐*MDA5* antibody‐positive CADM and rapidly progressive interstitial pneumonitis, whose disease was uncontrollable on intensive combined immunosuppressive therapy (glucocorticoids in combination with immunosuppressive agents), were treated with a dual therapy with increasing trough concentrations of tacrolimus (glucocorticoids combined with tacrolimus). Tacrolimus was administered orally at an initial dose of 0.075 mg/kg/day, and tacrolimus trough concentrations were measured daily 12 h after administration.

In this study, tacrolimus dosage adjustments were individualized based on patients′ clinical responses, changes in blood drug concentrations, and disease severity. For patients with more severe conditions, a faster dose escalation rate might be selected, while a slower rate was used for those with milder conditions. The specific escalation rate was flexibly adjusted according to patients′ treatment goals and clinical responses, thus no fixed escalation rate was established. Regarding the timing of achieving optimal concentrations, this varies among patients due to differences in disease status and individual responses. Tacrolimus concentrations typically require time to stabilize, and the duration of concentration changes is influenced by the patient′s condition and individual response. Therefore, during treatment, we flexibly adjust the dose based on each patient′s blood concentration trend and ensure the optimal dose is determined after concentration stabilization. When establishing the optimal Tacrolimus dose for each patient, we have fully considered the timing and trend of drug concentration changes to ensure the rationality of dose adjustments.

The tacrolimus trough concentration at the time of the initial improvement in the patient′s laboratory data (lower ferritin and lactate dehydrogenase levels and a lower oxygenation index) was considered the optimal trough concentration for that patient. The tacrolimus trough concentration was then individualized and adjusted during the following treatment period based on a combination of factors (the severity of the disease, the level of tacrolimus trough concentration, and adverse effects experienced by the patient).

Due to variations in treatment response among patients, we have not established a specific percentage reduction in biomarkers as the criterion for increasing concentrations. Instead, we adjust the Tacrolimus dosage based on each patient′s individual response to achieve optimal therapeutic outcomes.

The initial dose of glucocorticoids (methylprednisolone) was 1–2 mg/kg/day during dual therapy treatment, while hormone doses were adjusted at the physician′s discretion based on experience with appropriate treatment of DM. During the treatment period, cotrimoxazole was administered to all patients to prevent Pneumocystis jirovecii pneumonia. To ensure patient safety and minimize the risk of infection, stringent infection precautions were taken: maintaining the temperature and humidity of the wards at an appropriate level, strict hand hygiene management by healthcare staff before contact with patients and ensuring air circulation in the wards. Single‐occupancy wards were also provided.

### 2.5 Statistical Analysis

Statistical analyses were performed using SPSS software (version 25, IBM Corp., Armonk, NY, USA), and graphical visualizations were created with Prism 10.

## 3 Results

**Figure 1:**
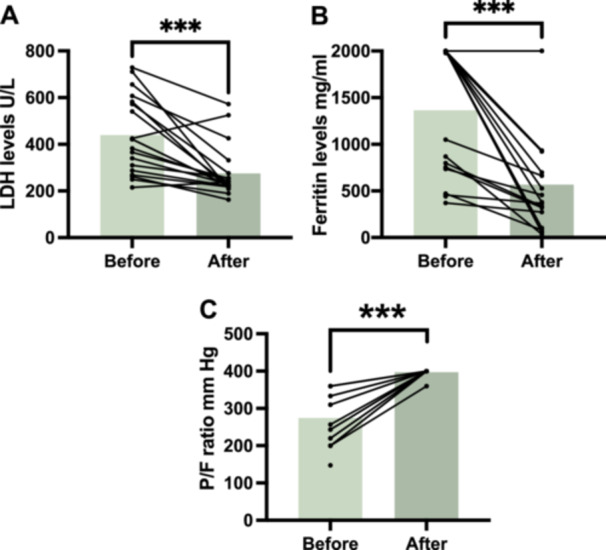
Changes in clinical biomarkers before and after tacrolimus treatment in patients with anti‐*MDA5* antibody‐positive CADM with rapidly progressive interstitial pneumonia. Paired comparisons were conducted for three key biomarkers pre‐ and posttreatment: (A) Serum lactate dehydrogenase (LDH) levels (U/L). (B) Ferritin levels (mg/mL). (C) Arterial oxygenation index (P/F ratio, mmHg). Significant improvements were observed in all parameters following tacrolimus therapy. Statistical analysis was performed using a paired *t*‐test or Wilcoxon signed‐rank test as appropriate. Asterisks indicate levels of statistical significance: *** *p* < 0.001.

Of the 19 patients that had refractory anti‐*MDA5* antibody‐positive CADM with rapidly progressive interstitial pneumonitis treated with a combination of increasing tacrolimus trough concentration‐escalating dual therapy, 16 displayed decreased ferritin and lactate dehydrogenase levels, an improved oxygenation index, and improved high‐resolution lung CT findings. However, two patients died after glucocorticoid therapy combined with tacrolimus therapy. One patient (Case 9) developed renal impairment on Day 14 of treatment when the tacrolimus trough concentration was 15.68 ng/mL. Lowering the tacrolimus trough concentration to 5.5 ng/mL resulted in a gradual return of the patient′s renal function to normal; however, because of the addition of cyclophosphamide due to disease activity, the patient did not experience the full efficacy of bimodal therapy during the treatment period. The patient′s clinical characteristics and disease duration are summarized in Table 1. Laboratory data indicated that ferritin and lactate dehydrogenase levels decreased, and the oxygenation index improved (p‐value of ferritin, lactate dehydrogenase levels, and oxygenation index < 0.01) after treatment compared with before treatment (Table 2 and Figure 1).

**Table 2:** Changes in biomarkers before and after treatment in patients with refractory *MDA5*‐antibody‐positive CADM with rapidly progressive interstitial pneumonia.

Items	Before treatment (median, IQR)	After treatment (median, IQR)	*p* value
LDH (U/L)	439.47 (164.56, 278.15)	275.30 (111.71, 46.15)	< 0.01
Ferritin (ng/mL)	1978 (703.31, 1396.5)	368 (586.24, 544.40)	< 0.01
P/F ratio (mm Hg)	283.5 (64.61, 145.0)	450 (22.5, 0.0)	< 0.01

*Note:* Paired comparisons were conducted for three key biomarkers pre‐ and posttreatment: (A) serum lactate dehydrogenase (LDH) levels (U/L), (B) ferritin levels (mg/mL), (C) arterial oxygenation index (P/F ratio, mmHg). Data were analyzed using a paired *t*‐test (for normally distributed data) and the Wilcoxon test (for non‐normally distributed data).

However, given the small sample size of this study (*n* = 19), we conducted a post‐hoc efficacy analysis. Cohen′s d effect sizes indicated substantial effects for LDH and ferritin (−1.20 and −1.38, respectively), but with extremely low power (1.20e−05 and 3.83e−06). The oxygenation index demonstrated a more significant effect (Cohen's *d* = 2.13, power = 0.59). Although power was low, it still detected a detectable effect.

**Figure 2:**
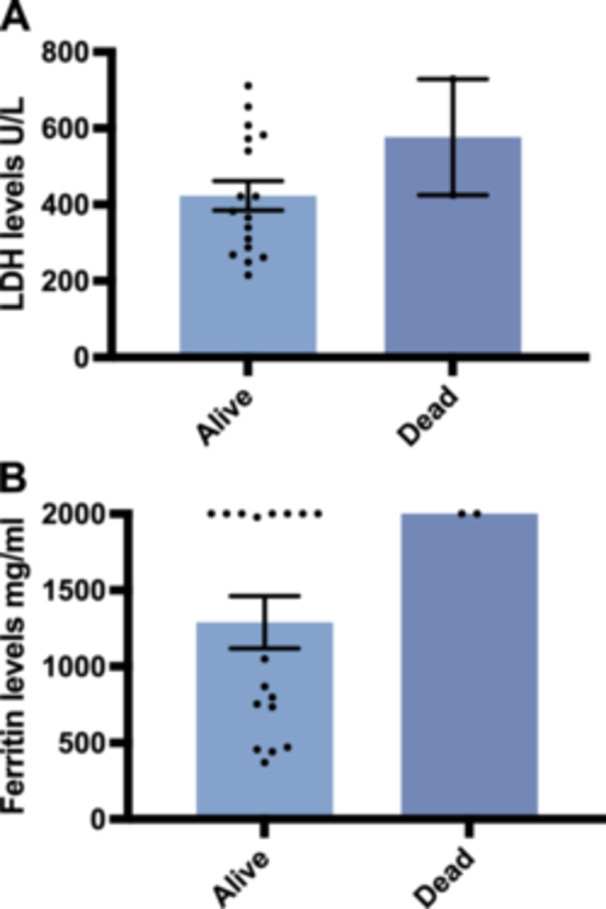
Baseline biomarker differences between survivors and non‐survivors among patients with anti‐*MDA5* antibody‐positive CADM with rapidly progressive interstitial pneumonia. This figure illustrates the comparison of key baseline biomarkers between patients who survived and those who died during follow‐up. (A) Serum lactate dehydrogenase (LDH) levels (U/L); (B) serum ferritin levels (mg/mL). There were no significant differences between the survivors and non‐survivors among patients in the indicators. Statistical analysis was performed using the Mann–Whitney *U* test.

**Figure 4:**
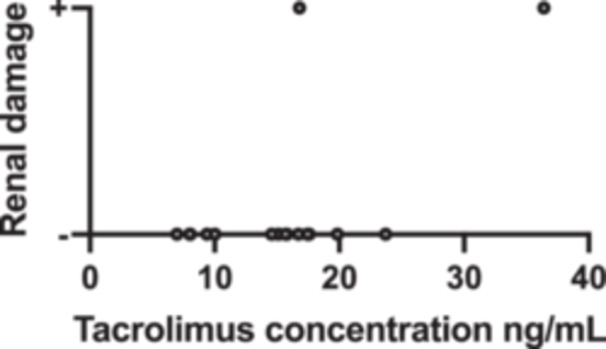
Relationship between Tacrolimus Concentration and Renal Damage in Patients with anti‐*MDA5* antibody‐positive CADM with rapidly progressive interstitial pneumonia. This scatter plot illustrates the correlation between whole‐blood tacrolimus trough concentrations (ng/mL) and the extent of renal impairment in patients diagnosed with anti‐*MDA5* antibody‐positive CADM with rapidly progressive interstitial pneumonia. Each data point represents an individual patient measurement.

Before treatment with tacrolimus trough concentration‐escalating bipartite therapy (glucocorticoids combined with tacrolimus), the data showed that there was no significant correlation between the need for mechanical ventilation and whether the patients survived (*p* value = 0.474) (Table 3). Additionally, the oxygenation index was significantly higher among the survivors compared with those who died (oxygenation index *p*‐value = 0.037), whereas there was no significant difference in ferritin and lactate dehydrogenase levels (ferritin level *p*‐value = 0.144, lactate dehydrogenase level *p*‐value = 0.160) (Table 4); box plots of the biomarkers are shown in Figure 2. Comparisons of the high resolution CT (HRCT) images of some patients before and after treatment (Figure 3) showed significant absorption of solid and ground glass shadows in the patients′ lungs after treatment. Of the 16 patients who improved, Case 17 (with an optimal trough concentration of 36.41 ng/mL) had an adverse reaction of renal impairment during the 6th week of treatment, which gradually normalized after lowering the tacrolimus trough concentration. Case 17 also developed cytomegalovirus reactivation and *Candida albicans* infection during treatment but responded well to antiviral and antifungal therapies without serious consequences. Of the two patients who died, one had an oxygenation index of 200 mmHg and mediastinal emphysema before tacrolimus trough concentration‐escalating dual therapy. This patient developed cytomegalovirus reactivation on Day 7 of treatment and died of respiratory failure on Day 11. The other patient had an oxygenation index of 147.5 mmHg before tacrolimus trough concentration‐escalating dual therapy and met the criteria for mechanical ventilation, before dying of respiratory complications on Day 4. In both patients, Spearman′s correlation analysis showed no significant correlation between tacrolimus concentration and renal damage (*p*‐value = 0.140) (Table 5), and the scatter plot of the correlation analysis is shown in Figure 4.

**Table 3:** Comparison of mechanical ventilation status between survivors and deceased with refractory *MDA5*‐antibody‐positive CADM with rapidly progressive interstitial pneumonia.

Item	Yes/no	Survive or not		Total	*p* value
		No	Yes		
Whether mechanical ventilation is required	No	0	9	9	0.474
	Yes	2	8	10	
Total		2	17	19	

*Note:* Data were analyzed using Fisher′s exact test.

**Table 4:** Comparison of clinical indicators between survivors and deceased with anti‐*MDA5* antibody‐positive CDAM with rapidly progressive interstitial pneumonia.

Items	Yes/no	Sample size	Median	Standard deviation	Statistical volume	*p*‐value	Median value difference	Cohen′s *d*
LDH(U/L)	Yes	17	382.8	157.969	6	0.144	193.9	0.948
	No	2	576.7	214.678				
	Total	19	421.9	164.562				
Ferritin(ng/mL)	Yes	17	1050	707.156	7	0.160	950	1.035
	No	2	2000	0				
	Total	19	1978	703.314				
Oxygenation index	Yes	17	310	65.377	32.5	0.037**	136.25	1.759
	No	2	173.75	37.123				
	Total	19	257	71.677				

*Note:* Data were analyzed using Mann–Whitney test. ***, **, * represent 1%, 5%, and 10% significance levels respectively.

Abbreviation: LDH, lactate dehydrogenase.

Figure 3Changes in chest CT findings of selected patients. HRCT images show that lung consolidation shadows and ground‐glass opacities are significantly absorbed in some patients after treatment.

**Figure:**
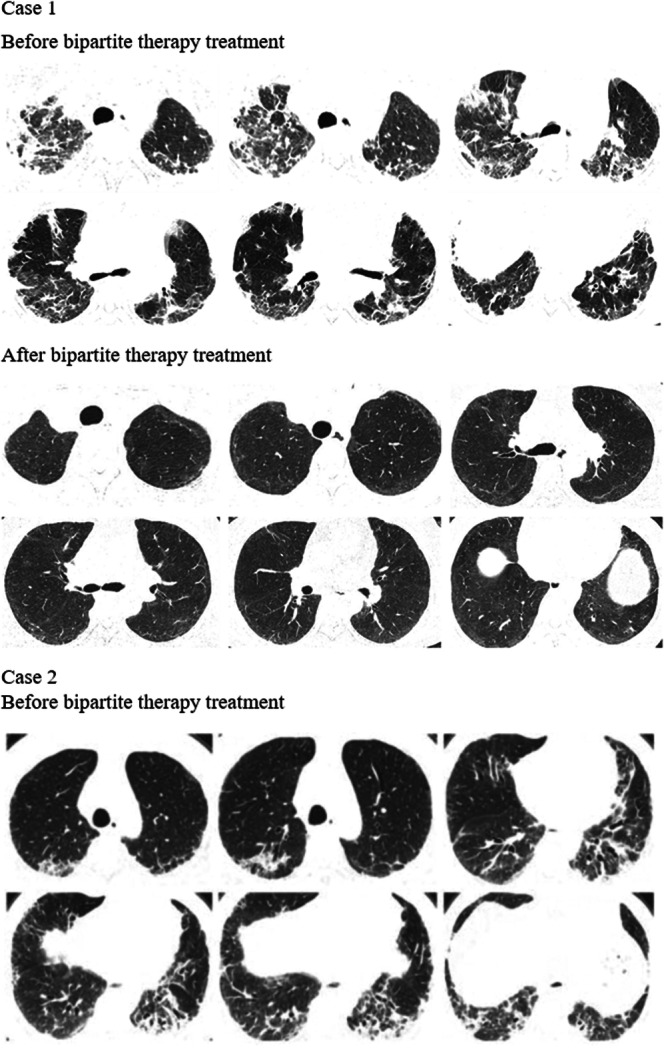

**Figure:**
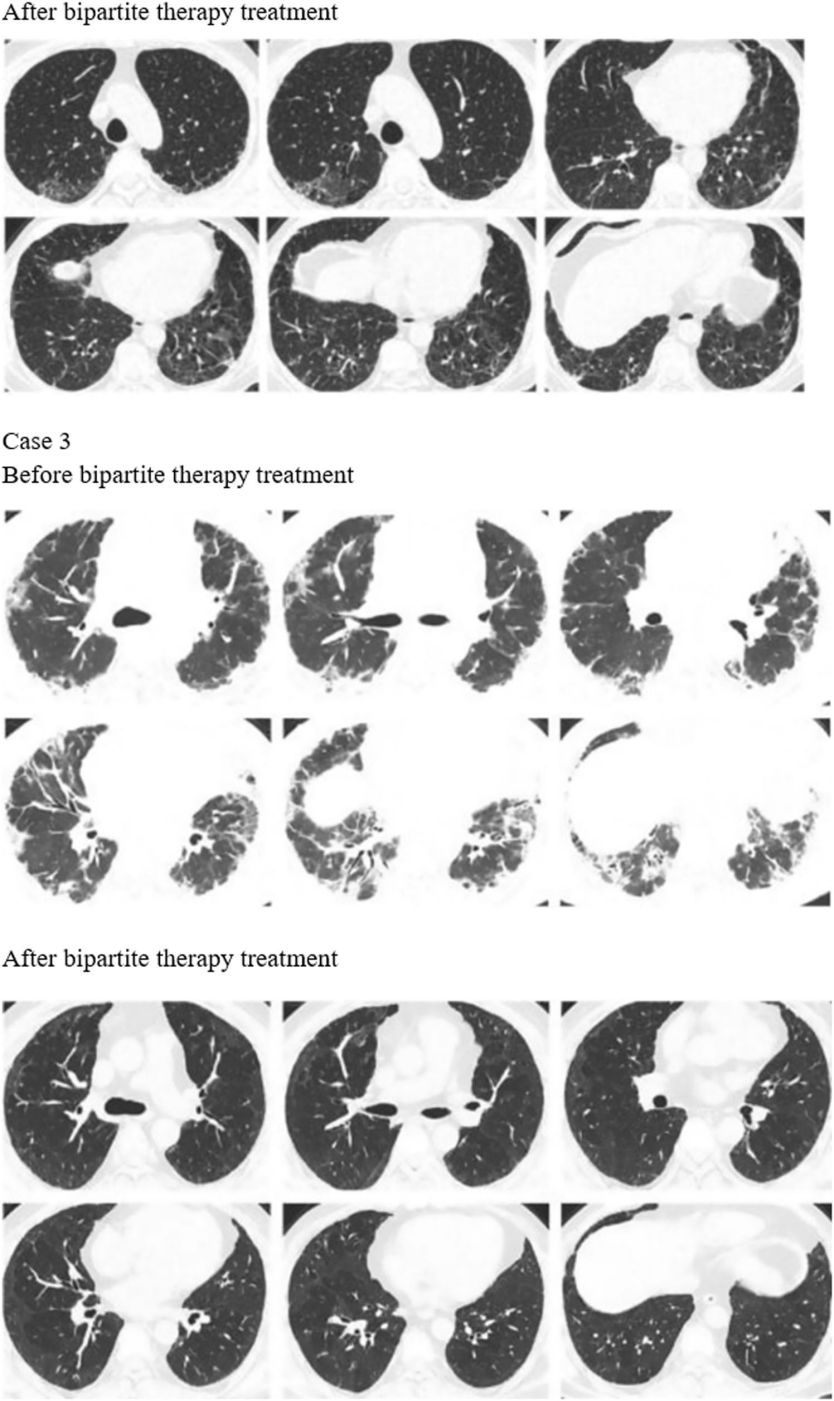

**Figure:**
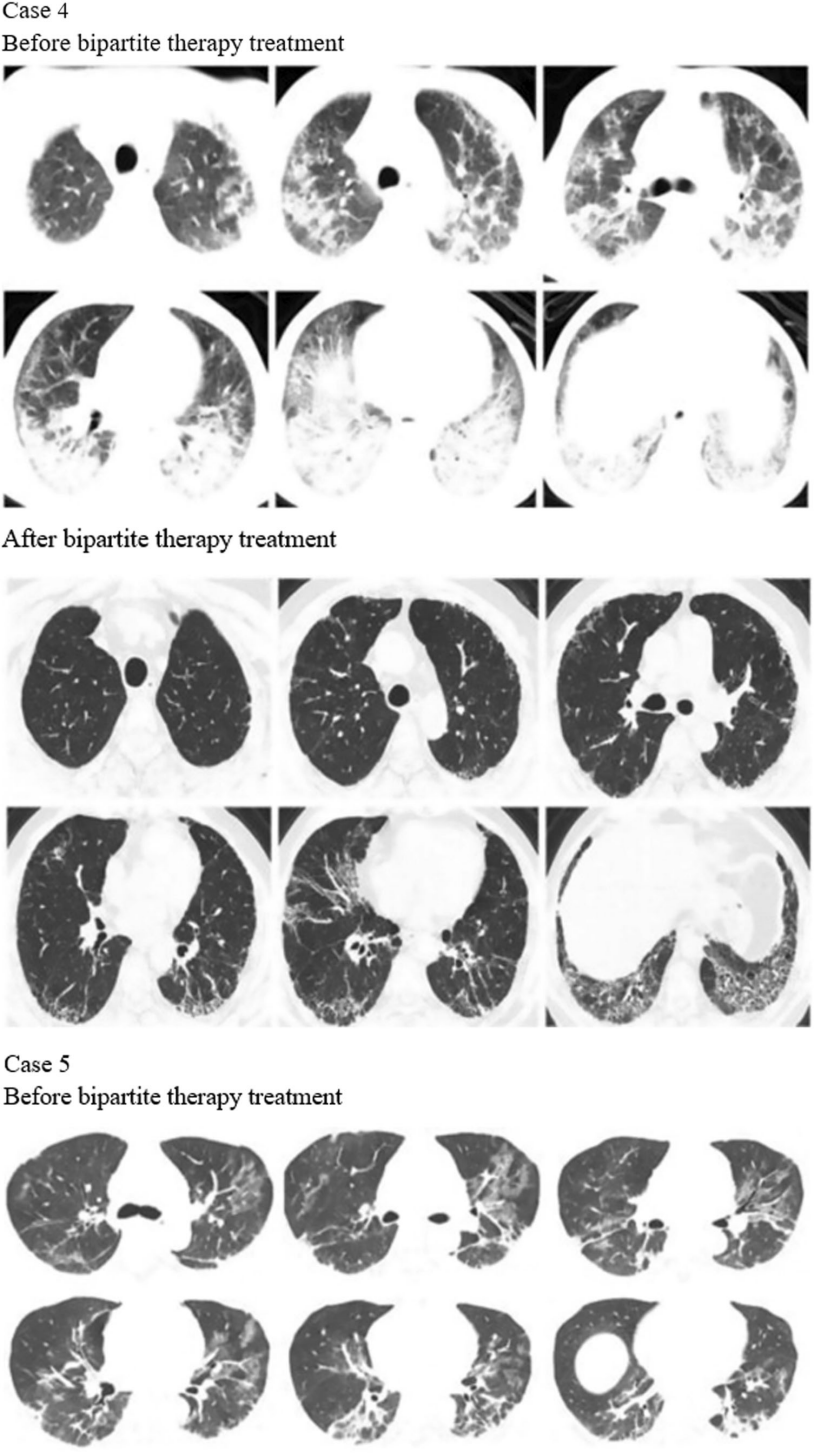

**Figure:**
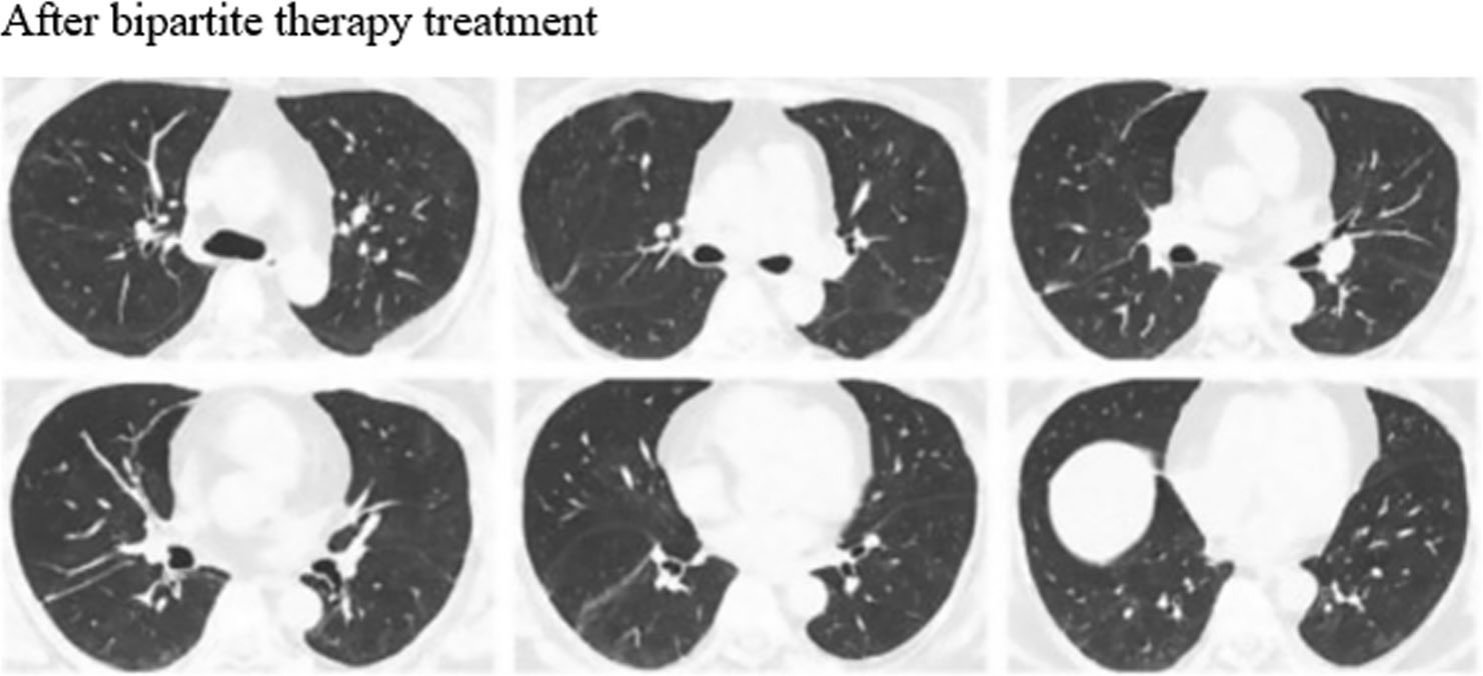

**Table 5:** Correlation between tacrolimus concentration and renal impairment in patients with refractory *MDA5*‐antibody‐positive CADM with rapidly progressive interstitial pneumonia.

	Tacrolimus trough concentration	Renal impairment
Tacrolimus trough concentration	1 (0.000***)	0.373 (0.140)
Renal impairment	0.373 (0.140)	1 (0.000***)

*Note:* Data were analyzed using paired *t*‐test or Wilcoxon's signed‐rank test as appropriate. ***, **, * represent 1%, 5%, and 10% significance levels respectively.

## 4 Discussion

A two‐drug combination of high‐dose corticosteroids plus a calcineurin inhibitor or a three‐drug combination of intravenous cyclophosphamide (IVCY) added to a two‐drug regimen is usually recommended as the treatment of choice [14]. However, while these conventional regimens have been shown to be effective in some patients, they have been shown to be ineffective in some refractory patients.

In this study, patients received initial treatments such as glucocorticoids and low‐dose tacrolimus, which are generally considered relatively mild. While these therapies achieved favorable outcomes in some patients, those with severe disease who did not respond to treatment were defined as “refractory.” This definition accounts not only for individual patient responses but also for clinical manifestations of poor treatment efficacy and inadequate disease control.

Currently, there is no unified treatment consensus for this disease, particularly regarding the selection of optimal first‐line therapy. Different clinicians and regions may choose varying treatment approaches based on individual patient conditions. Consequently, while glucocorticoids and tacrolimus are common treatment regimens, their efficacy varies significantly among patients and fails to effectively control the disease in certain individuals. Therefore, there is an urgent need for further research aimed at establishing unified treatment standards and optimal strategies.

Based on this, we enrolled refractory CADM patients who tested positive for anti‐melanoma differentiation‐associated gene 5 (*MDA5*) antibodies and presented with rapidly progressive interstitial pneumonia unresponsive to combined immunosuppressive therapy. Through studying these patients, we aim to provide additional clinical insights for treating refractory CADM.

Previous clinical trials have shown that tacrolimus has a dose‐dependent effect in patients with ulcerative colitis, with higher remission rates obtained after increasing the dose of tacrolimus administered. In a retrospective study in Japan, Suzuka et al. [15] treated two patients with DM and acute/subacute interstitial pneumonitis using glucocorticoids in combination with a high trough concentration of tacrolimus, with improvement in one patient and stabilization in the other. The patients included in our study achieved good outcomes after receiving tacrolimus trough concentration‐escalating bipartite therapy (glucocorticoids combined with tacrolimus), with 17 patients surviving and 2 patients dying.

Moreover, ferritin levels > 1000 ng/mL and an oxygenation index < 150 mmHg are strongly associated with high mortality and are often used as predictors of poor prognosis [16]. The patients we included with these poor prognostic factors were treated with tacrolimus trough concentration‐escalating bipartite therapy (glucocorticoids combined with tacrolimus), which resulted in a reduction in ferritin levels and an improvement in the oxygenation index. We believe that tacrolimus trough concentration‐escalating bipartite therapy (glucocorticoids combined with tacrolimus) improves the prognosis in this group of patients with poor prognostic factors. In our study, the target trough concentration of tacrolimus, which is usually 5–20 ng/mL for the treatment of DM [15, 17], was higher among our enrolled patients (6.97–36.41 ng/mL). No effective regimens have been determined for refractory anti‐*MDA5* antibody‐positive CADM with rapidly progressive interstitial pneumonia. Tacrolimus trough concentrations can be increased when the patient′s condition remains uncontrolled at a tacrolimus trough concentration of 20 ng/mL. More importantly, because different patients have different degrees of responsiveness to tacrolimus, the optimal trough concentration of tacrolimus is not the same, and there are significant individual differences. Thus, it is necessary to individually map out the optimal trough concentration of tacrolimus for each patient for better control of the disease during treatment. However, vigilance is needed to monitor the occurrence of tacrolimus‐related adverse events during dual therapy with increasing tacrolimus trough concentrations, especially when the trough concentration exceeds 20 ng/mL. Theoretically, the higher tacrolimus trough concentrations are usually associated with a higher risk of renal damage [18]. However, our study showed no significant correlation between tacrolimus concentration and renal damage, which may be due to our inclusion of a relatively small sample size and the presence of more pronounced individual differences. For example, in this study, one patient (Case 9) developed renal damage at a tacrolimus trough concentration of 15.68 ng/mL. Meanwhile, two other patients (Cases 15 and 17) developed renal damage at tacrolimus trough concentrations of 23.5 and 36.41 ng/mL, respectively, suggesting that renal damage is not only related to the tacrolimus trough concentration level [18] but also to differences in the response to tacrolimus [18]. For two patients who developed renal damage, after lowering the tacrolimus trough concentration, renal function gradually returned to normal. The optimal trough concentration in Case 17 was 36.41 ng/mL, which was maintained throughout the treatment. However, despite renal damage developing in the sixth week, the use of high tacrolimus concentrations helped them overcome the risky period of the disease. Therefore, whether tacrolimus‐associated renal damage occurs as an adverse effect is determined by a combination of factors such as differences in patient response to tacrolimus [18], the tacrolimus trough concentration [18], and the duration of use of high trough concentrations of tacrolimus. During tacrolimus trough concentration‐escalating dual therapy, both the increase and maintenance of tacrolimus trough concentration require close and dynamic monitoring of the renal function to adjust the tacrolimus trough concentration promptly and consequently maximize the restoration of renal function. In addition to the adverse effects of renal impairment, we observed cytomegalovirus reactivation and *C. albicans* infection in one patient (Case 17) at tacrolimus trough concentrations of 36.41 and 26.78 ng/mL, respectively. During tacrolimus trough concentration‐escalating dual therapy, strict infection protection measures, such as maintaining appropriate temperature and humidity in the ward, strict hand hygiene management by healthcare workers before contact with patients, and ensuring air circulation in the ward, should be implemented to prevent cytomegalovirus and fungal infection.

Furthermore, two patients died, one of whom had an oxygenation index of 147.5 mmHg, which fulfilled the criteria for mechanical ventilation, before treatment with tacrolimus trough concentration‐escalation dual therapy. The patient died of respiratory failure due to RPILD on day 4. Another patient with an oxygenation index of 200 mmHg before tacrolimus trough concentration incremental dual therapy met the criteria for mechanical ventilation combined with mediastinal emphysema and died of respiratory failure on day 11. Identifying an appropriate treatment strategy for refractory *MDA5*‐positive patients with very low oxygenation index and meeting the criteria for mechanical ventilation is challenging.

This study had some limitations owing to the nature of the case series. However, our experience suggests that in refractory anti‐*MDA5* antibody‐positive CADM with rapidly progressive interstitial pneumonitis, tacrolimus trough‐concentration‐escalating dual therapy is a therapeutic strategy to be considered in the absence of a definitive and effective treatment regimen. For each patient, the risks and benefits of tacrolimus trough escalation should be carefully evaluated.

## 5 Conclusion

Tacrolimus trough concentration‐escalating bipartite therapy (glucocorticoids combined with tacrolimus) and individualized mapping of the optimal tacrolimus trough concentration for each patient is a therapeutic strategy to be considered in patients with refractory anti‐melanoma differentiation‐associated gene 5 (*MDA5*) antibody‐positive clinically amyopathic dermatomyositis with rapidly progressive interstitial pneumonia. The exploration of the optimal trough concentration should be accompanied by close dynamic monitoring of the patient′s renal function and infection prophylaxis.

## Author Contributions

Anji Xiong and Jiang Shao were involved in the acquisition and interpretation of data and study conceptualization. Yiman Ye, Yanzao Zhao, Xi He, Jie Luo, and Xuemei Huang were involved in the editing of the manuscript. All authors approved the final version of the manuscript.

## Ethics Statement

Ethics approval was obtained from Nanchong Hospital of Beijing Anzhen Hospital Capital Medical University Privacy Office.

## Conflicts of Interest

The authors declare no conflicts of interest.
